# Recent Advances in Extended Ocular Drug Delivery for the Ocular Surface

**DOI:** 10.3390/molecules31111883

**Published:** 2026-05-31

**Authors:** Yura Choi, Mi-Young Jung, Eunsun Han, Choul Yong Park

**Affiliations:** Department of Ophthalmology, Samsung Medical Center, Sungkyunkwan University School of Medicine, Seoul 06351, Republic of Korea; ychoi.op@gmail.com (Y.C.);

**Keywords:** eye, drug, biopolymer, drug delivery, platform, ocular surface

## Abstract

The unique anatomy and physiological barriers of the human eye—particularly rapid tear turnover and limited corneal permeability—present significant obstacles to achieving effective topical drug delivery. In response to these constraints, biopolymer-based extended-release systems have emerged as a promising and transformative class of ocular therapeutics. This review provides a comprehensive overview of recent advances in natural biopolymers, including polysaccharides and protein-derived polymers, for application on the ocular surface. These materials exhibit advantageous characteristics such as mucoadhesion, biocompatibility, and stimuli-responsive behavior, which collectively enhance precorneal residence time and enable controlled, sustained drug release. We further discuss diverse delivery platforms—ranging from in situ forming hydrogels and mucoadhesive nanoparticles to drug-eluting contact lenses and microneedle-based systems. In addition, we highlight how the integration of nanotechnology and bioinspired scaffolds can augment the delivery efficiency of therapeutic agents to ocular tissues. Overall, this review underscores the ongoing transition from conventional topical eye drops to sophisticated, functionalized delivery systems capable of maintaining therapeutic drug levels while simultaneously supporting tissue repair and wound healing. Finally, we outline the remaining challenges in clinical translation and consider the future potential of smart, responsive biopolymer systems in advancing the treatment of both anterior and posterior segment diseases.

## 1. Introduction

The human eye is a highly sophisticated sensory organ with a unique anatomy that provides a high level of protection against external input, however, this same sophistication creates significant challenges for drug delivery [1,2]. While topical administration is the preferred and least invasive route for treating anterior segment disorders, it is plagued by low bioavailability, typically less than 5% [3]. The primary obstacles to effective topical delivery are static barriers (the cornea, conjunctiva, and sclera) and dynamic barriers (tear turnover, blinking reflex, and nasolacrimal drainage) [4]. Rapid tear turnover and blinking result in the loss of the majority of topically applied drugs within minutes [5]. For instance, the lacrimal turnover rate is approximately 1 µL/min, meaning most eye drop solutions are washed away within minutes of instillation [6,7]. To compensate, frequent high-dose administrations are required, leading to poor patient compliance and potential local or systemic side effects as the drug contacts eyelid or enters the bloodstream through the nasolacrimal duct [8].

Consequently, there is an urgent clinical demand for extended drug delivery systems that can bypass these physiological hurdles, enhance precorneal residence time, and maintain therapeutic drug levels over prolonged periods [9]. In this context, biopolymers have emerged as the cornerstone of ocular pharmaceutical innovation [8,10,11,12]. Unlike traditional formulations, biopolymers offer tunable physicochemical properties—such as mucoadhesion, stimuli-responsiveness, and controlled degradation—that are essential for overcoming the eye’s clearance mechanisms [11].

Historically, the safety and efficacy of biopolymers in ophthalmology have been proven through the decades-long success of contact lenses and intraocular lenses. However, recent advances have shifted the focus from purely structural or refractive uses toward functionalized drug-eluting platforms (Table 1). Modern research now integrates bio-inspired materials, such as collagen and gelatin, with advanced fabrication techniques like electrospinning, 3D bioprinting, and nanotechnology [10,13]. These biopolymer-based systems, including in situ forming hydrogels, mucoadhesive nanoparticles, and drug-eluting ocular inserts, enable a more personalized therapeutic strategy for the ocular diseases [14]. By mimicking the extracellular matrix and providing high drug-binding capacity, these materials not only extend therapeutic exposure but also can promote corneal epithelial regeneration and wound healing in diseased conditions [15,16,17].

To ensure transparency and provide a balanced overview of the current landscape, a comprehensive literature search was performed. Primary databases, including PubMed/MEDLINE, Web of Science, and Google Scholar, were searched to identify relevant studies published primarily between January 2010 and May 2024, though seminal earlier works were included for historical context.

The search strategy incorporated targeted keywords and Boolean operators to capture the multidisciplinary scope of the field. Terms were grouped into (1) biopolymer classes (e.g., chitosan, hyaluronic acid, silk fibroin, cellulose derivatives), (2) delivery architectures (e.g., in situ gels, drug-eluting contact lenses, microneedles), and (3) functional mechanisms (e.g., mucoadhesion, stimuli-responsiveness, degradation-controlled release). Additional keywords related to clinical indications—such as dry eye disease, glaucoma, and corneal regeneration—were included to identify studies with strong translational relevance.

Inclusion criteria focused on peer-reviewed original research and clinical reports that demonstrated significant advancements in (1) enhancing precorneal residence time, (2) improving ocular bioavailability, and (3) utilizing natural or hybrid biopolymers for drug delivery platforms. Exclusion criteria were applied to studies that focused solely on non-biopolymer synthetic materials without biological integration, or those lacking clear evidence of ophthalmic application. By following this approach, we minimized selection bias and provided a representative analysis of the most impactful recent innovations in the field.

**Figure 1:**
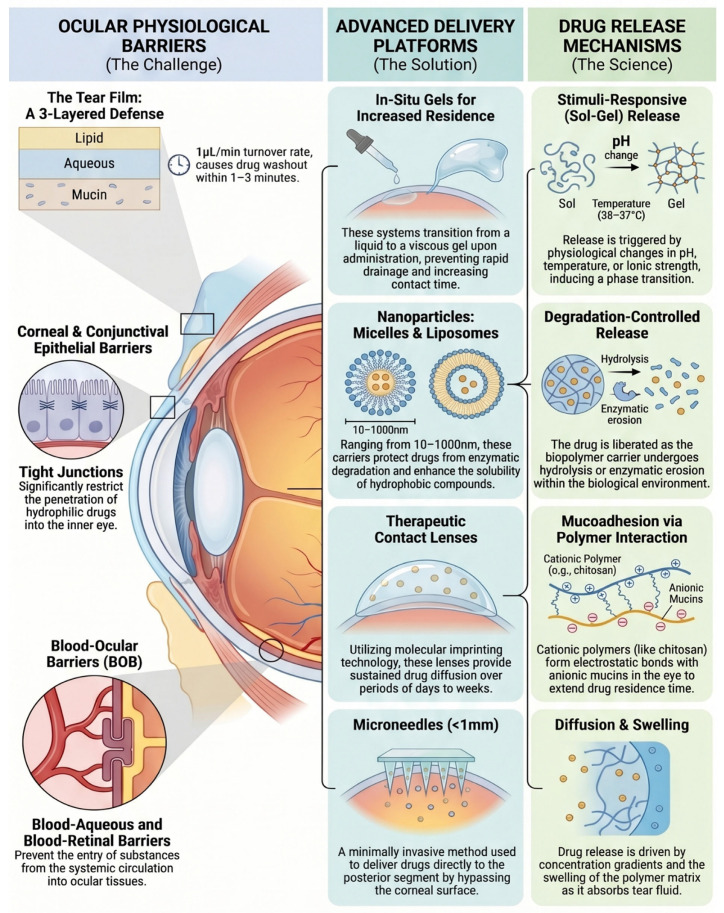
Overview of Ocular Surface Barriers and Biopolymer-Based Strategies for Enhanced Drug Delivery.

This review aims to provide a comprehensive overview of recent advancements in biopolymer-based extended drug-delivery systems specifically designed for ocular surface applications. We introduce the major biopolymers utilized in this field, examine the diverse delivery platforms developed to enhance ocular drug penetration, and discuss strategies for achieving controlled and sustained release (Figure 1). In addition, we summarize the latest progress in ocular biopolymer research and offer perspectives on future directions [8,10,18].

This schematic illustrates the major physiological barriers that limit ocular drug delivery, including the tear film, the corneal and conjunctival epithelia, and the blood–ocular barriers. To address these challenges, a range of delivery platforms has been developed, such as in situ gelling systems that prolong residence time, nanoparticles (including micelles and liposomes) that protect drugs from enzymatic degradation, therapeutic contact lenses enabling sustained release, and microneedles that facilitate minimally invasive delivery to the posterior segment. The diagram also highlights four principal biopolymer-mediated drug-release mechanisms: mucoadhesion driven by electrostatic interactions, diffusion- and swelling-based release governed by concentration gradients, stimuli-responsive release involving sol–gel transitions, and degradation-controlled release through hydrolysis or enzymatic erosion. Figure 1 was created using NotebookLM.

## 2. Biopolymers for Extended Ocular Drug Delivery

Biopolymers serve as essential building blocks in extended ocular drug delivery systems because they offer biocompatibility, biodegradability, and adaptable physicochemical properties [9,10,12]. These materials are engineered to overcome the eye’s natural protective barriers by enhancing precorneal residence time and providing matrices capable of sustained therapeutic release [10,11]. Among the various biopolymer categories, polysaccharide-based materials and protein-based polymers are both widely utilized, each contributing unique characteristics that influence their performance in ocular applications [19,20,21].

Protein-derived polymers—including collagen, silk fibroin, gelatin, elastin-like polypeptides, and albumin—are composed of amino-acid sequences that can interact favorably with ocular tissues [19,22,23]. Their degradation typically yields small peptides or amino acids, and their structural similarity to endogenous proteins supports compatibility with the corneal and conjunctival environment [19,20,21]. Polysaccharide biopolymers such as hyaluronic acid, chitosan, alginate, and dextran originate from natural carbohydrate sources and are also broadly recognized as safe [19,20,21]. However, their biological interactions may vary depending on molecular charge, source, and degree of purification, and their enzymatic degradation profiles can differ from those of protein-based systems [19,22,23].

Maintaining optical clarity is essential for any material intended for direct corneal contact, since the preservation of transparent light transmission is fundamental for normal visual function [24]. Protein-based materials can be processed into films and hydrogels with high transparency, while polysaccharide systems exhibit a wider range of optical properties depending on formulation and molecular structure [19,22,23]. Protein polymers allow modulation of stiffness and degradation through adjustments in secondary structure or crosslinking density, whereas polysaccharide materials typically rely on enzymatic or ionic mechanisms for structural modification, which may offer different degrees of control [19,22,23].

**Figure 2:**
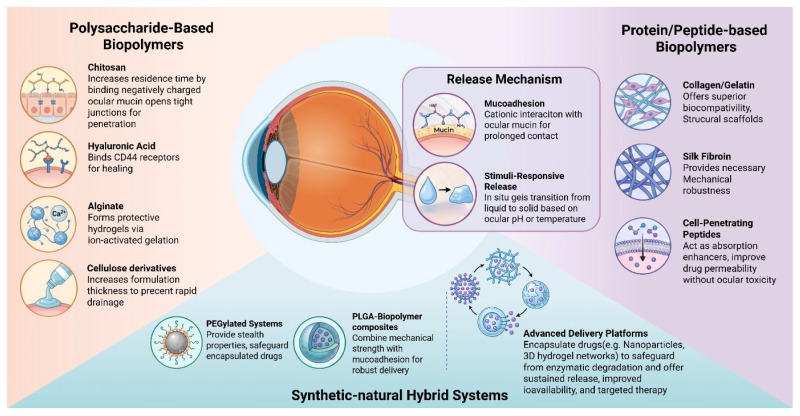
Classification and Functional Mechanisms of Biopolymers in Ocular Drug Delivery.

Both polymer types can serve as carriers for therapeutic agents [25,26,27]. Polysaccharides are effective for stabilizing and delivering hydrophilic small molecules, while protein-based matrices can create environments that help preserve the structural integrity of biologics—such as peptides, antibodies, and nucleic acids—by forming hydrogen-bonding networks that protect them from denaturation and enzymatic degradation [19,22,23]. Additionally, protein polymers may contain intrinsic motifs that support cell adhesion, whereas polysaccharide materials generally require chemical modification to introduce similar bioactive features [19,22,23] (Figure 2).

Although lipids are also important biomaterials used in drug delivery, their application to the ocular surface is typically limited to being used as standalone nanocarriers or as hybrid systems in which they coat the surface of a main biopolymer composed of polysaccharides or peptides to enhance drug-delivery efficiency.

Biopolymers are categorized into three primary classes based on their origin and composition: polysaccharide-based (e.g., chitosan, hyaluronic acid, alginate), protein/peptide-based (e.g., collagen, silk fibroin, cell-penetrating peptides), and synthetic-natural hybrid systems (e.g., PEGylated or PLGA-composites). These materials facilitate enhanced therapeutic outcomes through distinct release mechanisms, such as mucoadhesion via cationic interactions with ocular mucin and stimuli-responsive release, where in situ gels undergo phase transitions triggered by ocular pH or temperature. Specific functionalities include the modulation of corneal tight junctions by chitosan, CD44 receptor-mediated attachment by hyaluronic acid, and the use of cell-penetrating peptides as non-toxic absorption enhancers. These advanced delivery platforms, ranging from nanoparticles to 3D hydrogel networks, safeguard encapsulated drugs against enzymatic degradation while providing sustained release and improved bioavailability for targeted ocular therapy. Figure 2 was created in NotebookLM and BioRender. Jung, M. (2026) https://BioRender.com/hqji8mk (accessed on 29 April 2026).

### 2.1. Polysaccharides Based Biopolymers

Polysaccharides are versatile natural biopolymers extensively utilized in ocular drug delivery due to their exceptional biocompatibility, biodegradability, and mucoadhesive properties (Table 2). Derived from diverse natural sources, these carbohydrate-based polymers offer a useful backbone for controlled release, improving precorneal residence time and therapeutic efficacy while minimizing ocular irritation and systemic side effects.

#### 2.1.1. Chitosan

Chitosan, a naturally derived cationic polysaccharide obtained from chitin deacetylation, has attracted significant attention for its unique combination of mucoadhesive, biocompatible, and permeation-enhancing properties [15,18,28]. Chitosan possesses several physicochemical and biological properties that make it highly advantageous for ocular drug delivery [18,28,29]. Its cationic nature at physiological pH enables strong electrostatic interactions with negatively charged mucins on the ocular surface, providing notable muco-adhesion and prolonging precorneal residence time while reducing drug loss through tear drainage [29]. In addition, chitosan can transiently open tight junctions between corneal epithelial cells, enhancing paracellular permeability and thereby improving the penetration of hydrophilic drugs that typically exhibit poor corneal uptake [16,17]. Chitosan is also biocompatible, biodegradable, and exhibits low immunogenicity, supporting its safe use for repeated or long-term ocular applications [18]. The polymer’s intrinsic antimicrobial activity further augments the efficacy of antibiotic-loaded formulations, making them promising candidates for treating bacterial keratitis and for postoperative prophylaxis [30]. Furthermore, chitosan’s chemical structure allows extensive modification—such as trimethylation, thiolation, or carboxymethylation—enabling precise control over solubility, charge density, and drug-release behavior, and facilitating the development of highly tailored delivery systems [16]. Sustained-release chitosan carriers for antiglaucoma medications such as timolol, brimonidine, and latanoprost have shown encouraging results in maintaining intraocular pressure reduction over extended periods [29,31]. Additionally, chitosan-based systems have been investigated for the delivery of antioxidants and neuroprotective molecules—including curcumin, resveratrol, and nerve growth factor—to support retinal and optic nerve protection [32,33,34,35].

Chitosan has been integrated into a wide variety of ocular delivery platforms, including nanoparticles, hydrogels, coated systems, drug-eluting contact lenses, and microneedle-based technologies [16]. Chitosan nanoparticles and related nanocarriers have been extensively investigated for the sustained delivery of anti-inflammatory, antimicrobial, antiglaucoma, and antioxidant agents [36]. Their nanoscale size enhances corneal penetration, while the polymeric matrix provides controlled and prolonged drug diffusion [29]. Among these systems, chitosan–tripolyphosphate nanoparticles prepared through mild ionic gelation are particularly noteworthy. In addition, chitosan-coated liposomes or micelles can further improve mucoadhesion while preserving the inherent advantages of lipid-based carriers [29,37,38].

Thermosensitive or pH-responsive chitosan hydrogels have also been investigated (Table 3). These formulations undergo a sol–gel transition upon topical application to the ocular surface and can sustain drug release for several hours to several days, depending on their composition [31,39]. Furthermore, coating conventional nanoparticles or ocular implants with chitosan enhances surface charge, increases mucoadhesion, and modulates release kinetics—features that are especially beneficial for hydrophobic drugs encapsulated in non-chitosan matrices [16,29].

Chitosan has also been incorporated into, or applied onto, hydrogel contact lenses to create drug-eluting lenses capable of markedly prolonged release compared with topical eye drops, offering a promising strategy for chronic ocular diseases such as dry eye and glaucoma [40]. More recently, chitosan-based microneedle systems have emerged as an innovative platform for transscleral or intracorneal drug delivery, enabling the bypass of surface barriers and providing sustained release within deeper ocular tissues [41].

While chitosan is widely recognized for its strong mucoadhesiveness and biocompatibility, several limitations hinder its clinical translation for ocular surface drug delivery. Its poor solubility at physiological pH (7.4) often requires mildly acidic formulations, which can cause ocular irritation and reflex tearing [42]. Unmodified chitosan also exhibits high concentration-dependent viscosity, leading to transient blurred vision and reduced patient compliance. Moreover, despite its strong electrostatic attraction to the negatively charged mucin layer, rapid tear turnover can still wash it away unless chemically modified (e.g., thiolated or quaternized forms) [29].

**Table 3:** Preclinical Chitosan Hydrogels for Ocular Surface Drug Delivery.

Drug	Administration Route	Target Disease	Cross-Linking Method & Characteristics	Performance & Results	References
Latanoprost	Topical	Glaucoma	thermosensitive chitosan/gelatin/glycerol phosphate hydrogel	IOP was decreased within 7 days and was maintained within a normal range by a weekly topical administration.	[43]
Levofloxacin	Topical	Infection	thermosensitive hexanoyl glycol chitosan hydrogel	Aqueous concentration of levofloxacin doubled at 2 and 4 h after topical application with the hydrogel compared to solution.	[44]
Recombinante human nerve growth factor (rhNGF)	Topical	Keratitis	UV crosslinking of chitosan and azidobenzoic acid conjugate	rhNGF was released gradually over 24 h	[45]
Platelet rich plasma (PRP)	Topical	Keratitis	thermosensitive chitosan/glycerol phosphate hydrogel	Hydrogels containing PRP were successfully fabricated and non-toxic	[46]

#### 2.1.2. Hyaluronic Acid

Hyaluronic acid (HA) possesses several intrinsic properties that make it highly attractive for ocular drug delivery application [8]. Its strong water-binding capacity and viscoelastic nature enable HA to form hydrogels that adhere to the ocular surface and resist rapid clearance by tear flow, thereby prolonging precorneal residence time [19,22]. HA also interacts favorably with ocular tissues due to its natural presence in the cornea, conjunctiva, and vitreous, contributing to excellent biocompatibility and minimal immunogenicity [47]. Accordingly, HA is extensively used in ophthalmology for various applications, including dry eye treatment, contact lens comfort agents, vitreous substitutes, corneal wound healing, and ophthalmic viscoelastic devices [5,48,49].

In ocular surface drug-delivery systems, HA enhances ocular penetration primarily by extending residence time and facilitating diffusion through hydrated polymer networks, rather than by directly disrupting epithelial tight junctions [8,23,50,51,52]. Its chemical structure is highly amenable to modification—such as thiolation, methacrylation, or conjugation with therapeutic agents—allowing precise control over gelation behavior, degradation profiles, and drug-release kinetics [53,54,55].

Building on these advantages, a wide range of HA-based ocular surface drug-delivery systems has been developed [8]. HA hydrogels and in situ gelling formulations utilize temperature- or pH-responsive sol–gel transitions to form viscoelastic matrices on the ocular surface, enabling sustained drug release for periods ranging from hours to days [56] (Table 4). HA nanoparticles and drug–polymer conjugates have also been engineered to provide controlled release and enhanced tissue penetration, with the added advantage of potential CD44-mediated targeting [57,58] (Table 5 and Table 6). In addition, HA has further been incorporated into, or coated onto, silicone hydrogel contact lenses to create drug-eluting lenses capable of releasing therapeutic agents over extended durations while simultaneously improving lens wettability and comfort [59,60]. More recently, HA-based microneedles and injectable hydrogels have emerged as innovative platforms offering sustained drug release even to posterior segment tissues [61,62].

These HA-based extended-release systems have been investigated across a broad spectrum of ocular diseases [25,27]. HA hydrogels, nanoparticles and contact lens have been used to deliver cyclosporine, corticosteroids, lubricants, and anti-glaucoma medication [28,59,63]. In addition, HA has been widely reported to promote cornea epithelial healing, provide ocular surface lubrication, and modulate inflammation, thereby supporting improved therapeutic outcomes in bacterial keratitis [8,30,64]. HA carriers have also been applied to antioxidants and neuroprotective agents such as curcumin, resveratrol, and nerve growth factor to support retinal and optic nerve protection [32,33,34,35].

Despite these promising results, several challenges continue to limit the clinical translation of HA-based ocular delivery systems [22,65]. Variability in molecular weight, purity, and degree of modification can affect reproducibility and performance [22]. HA’s inherently limited mechanical strength and susceptibility to rapid enzymatic degradation may also restrict its suitability for long-term applications unless appropriately modified [23]. In addition, some formulations require acidic conditions or high polymer concentrations that may induce ocular irritation [66]. Manufacturing complexity and regulatory hurdles associated with polymer-based delivery platforms further impede commercialization. Addressing these issues will be essential for broader clinical adoption [65].

**Table 4:** Preclinical Hyaluronic Acid Hydrogels for Ocular Surface Drug Delivery.

Drug	Administration Route	Target Disease	Cross-Linking Method & Characteristics	Performance & Results	References
Latanoprost ester	Subconjunctival	Glaucoma	Hexamethylene diisocyanate-functionalized	Sustained release for 152 days in rabbit aqueous humor.	[67]
Ketoconazole	Topical	Keratitis	Poly(*N*-isopropylacrylamide)/HA	Transitions to gel at 33 °C; moderate release without burst effect.	[56]
Curcumin Nanoparticles	Topical	Keratitis	cyclodextrin encapsulation + HA matrix	Enhanced healing of ulcerative keratitis and reduced medication frequency.	[68]
5-Fluorouracil (5-FU)	Subconjunctival	Fibrosis	PLGA microspheres + HA hydrogel	Retarded drug release for 15 days to prevent ocular fibrosis.	[69]

**Table 5:** Preclinical Hyaluronic Acid -Based Nanocarriers for Ocular Surface Drug Delivery.

Drug	Administration Route	Target Disease	Carrier Type	Performance & Results	References
Ciprofloxacin	Topical	Infection	Zein/HA Nanoparticles	High encapsulation efficiency with sustained release over 24 h.	[70]
Latanoprost	Topical	Glaucoma	HA-Chitosan Nanoparticles	Achieved 29% IOP reduction, superior to Xalatan (23%).	[71]
Epoetin beta (EPO)	Topical	Glaucoma	Chitosan/HA Nanoparticles	Detected in the retina for up to 21 days post-administration.	[72]
Imatinib	Topical	Neovascularization	HA-ethylenediamine-hexadecyl Micelles	Facilitated corneal penetration and inhibited endothelial germination.	[73]
Genistein	Topical	Neovascularization	MPEG-PAE-g-HA Micelles	Delayed drug release and enhanced corneal penetration by 1.5x.	[74]
Timolol & Dorzolamide	Topical	Glaucoma	HA-modified Chitosan Nanoparticles	Duration of drug effect increased from 8 h to 12 h.	[75]

**Table 6:** Preclinical Hyaluronic Acid Surface Modification Nanocarriers for Ocular Surface Drug Delivery.

Carrier Bulk	Drug	Administration Route	Type of HA Coating	Modification Result & Performance	References
Gold NPs (AuNPs)	Ophthalmic drugs	Topical/Intravitreal	Covalent/Electrostatic	Doubled distribution in the posterior segment vs. uncoated particles.	[57,58]
Gelatin NPs	Epigallocatechin gallate	Topical	Electrostatic adsorption	Accumulated in cytoplasm; effectively treated dry eye syndrome.	[76]
Chitosan NPs	Dexamethasone	Topical	Electrostatic adsorption	Bioavailability was 2.14 times higher than drug solutions.	[77]
PCL NPs	Cyclosporine A	Topical	Electrostatic/Physical	Achieved corneal drug levels 1.5–1.9× higher than uncoated NPs.	[78]

#### 2.1.3. Alginate

Alginate is a natural polysaccharide extracted from brown seaweed [19,20,21]. Alginate consists of β-D-mannuronic acid (M) and α-L-guluronic acid (G) residues arranged in varying sequences [19,20,21]. The G-blocks interact strongly with divalent cations (e.g., Ca^2+^) to form the well-known “egg-box” structure, enabling rapid gelation under physiological conditions [22,23]. Its most significant property is the ability to undergo ion-activated in situ gelation [22,23]. Upon contact with the eye, it reacts with divalent cations like calcium (Ca^2+^) present in tear fluid to transform from a liquid drop into a viscoelastic gel [22,23]. This transition significantly extends precorneal residence time [25,26]. Alginate is well-tolerated and mucoadhesive, as its polymer chains swell and form non-covalent bonds with the mucin layer [66]. Formulations with high guluronic acid content are particularly effective for immediate gelation upon administration [22].

Alginate-based ocular delivery systems encompass nanoparticles, in situ gelling formulations, hydrogels, and ocular inserts, all designed to prolong drug residence time and achieve sustained release [25,26,27]. Alginate nanoparticles have been widely investigated for enhancing corneal penetration and extending therapeutic duration; for example, betamethasone-loaded alginate nanoparticles provided prolonged release and improved drug stability, while timolol-loaded nanoparticles produced longer-lasting intraocular pressure reduction than free timolol, and alginate–chitosan hybrid nanoparticles further increased mucoadhesion and ocular surface retention [29,31]. Ion-activated in situ gels represent another successful platform, where alginate solutions gel upon contact with tear-film Ca^2+^, forming viscoelastic matrices that adhere to the cornea; ciprofloxacin-loaded alginate in situ gels maintained antimicrobial activity for up to 24 h, and combinations with hydroxypropyl methylcellulose (HPMC) or poloxamers enhanced gel strength and controlled release [31,39]. Alginate hydrogels offer a hydrated, stable matrix suitable for both hydrophilic and hydrophobic drugs, with dexamethasone-loaded hydrogels showing multi-day controlled release and injectable alginate hydrogels demonstrating potential for long-term subconjunctival anti-inflammatory therapy [27,39]. Additionally, alginate-based ocular inserts and films provide predictable, prolonged release profiles, as antibiotic-loaded films sustained drug delivery for 48–72 h and improved outcomes in keratitis models, while bilayer inserts incorporating both fast- and slow-release layers enabled immediate and extended delivery within a single device [40].

Despite its advantages, alginate-based ocular delivery systems still face several challenges, including variability in molecular weight and M/G ratio that affects formulation reproducibility, rapid dissolution in tear fluid that limits long-term retention unless chemically or physically modified, and inherent mechanical weakness that often necessitates blending with supportive polymers such as chitosan or HPMC [22,23]. Additionally, Ca^2+^-mediated crosslinking can be unstable due to ion exchange with tear electrolytes, leading to premature gel weakening [22].

#### 2.1.4. Cellulose Derivatives

Cellulose is an abundant natural polymer that requires chemical modification for pharmaceutical use due to its crystalline structure and insolubility [19,20,21]. Despite its advantages, the crystalline structure of cellulose makes it inherently insoluble and non-fusible in most organic solvents, limiting its direct use in biomedical and pharmaceutical applications [19,20,21]. This challenge can be overcome by producing cellulose derivatives through chemical modifications such as esterification, etherification, or oxidation [22,23]. Common derivatives used as viscosity-enhancing agents in ophthalmic drops include methylcellulose (MC), hydroxypropyl methylcellulose (HPMC), carboxymethylcellulose (CMC), and hydroxyethyl cellulose (HEC) [19,22,23]. These polymers break down through enzymatic hydrolysis and possess mucoadhesive properties that increase the contact time of eye drops with the corneal surface [66]. By reducing the drainage rate, these derivatives significantly improve drug absorption and bioavailability [25,26].

Cellulose derivatives represent one of the most versatile classes of biopolymers used in extended ocular drug delivery, owing to their tunable physicochemical properties, biocompatibility, and ability to enhance drug retention on the ocular surface [19,20,21] (Table 7). In nanoparticle systems, cellulose derivatives act as stabilizers and solubility enhancers, particularly for poorly soluble drugs [29]. Among these, HPMC is widely incorporated into ophthalmic formulations as a viscosity enhancer, in situ gelling agent, and matrix former for sustained-release inserts [22,23]. CMC, another extensively used derivative, exhibits strong mucoadhesive properties and high water solubility, making it a key component in artificial tears and ocular drug carriers [66]. MC, known for its thermoresponsive behavior, forms gels at physiological temperature and is frequently employed in in situ gelling systems, ocular inserts, and lubricant formulations [25,26]. EC, a hydrophobic cellulose derivative, is commonly used to fabricate controlled-release membranes, microcapsules, and ocular films [22,29]. HEC and hydroxypropyl cellulose (HPC) also play important roles as viscosity enhancers, stabilizers, and film-forming agents. HPC-based inserts have provided sustained release for up to 24–48 h, while HEC has been shown to improve the stability and release profiles of hydrophobic drugs [29].

Overall, cellulose derivatives offer several important advantages for extended ocular drug delivery, including improved solubility of hydrophobic drugs, prolonged ocular residence through viscosity modulation and mucoadhesion, excellent biocompatibility, and broad versatility across diverse formulation platforms [19,20,21]. Their capacity for chemical modification further allows precise tuning of release kinetics and mechanical properties [22,23]. Despite these strengths, several challenges remain [14]. Certain derivatives exhibit insufficient mechanical robustness, and variability in viscosity grades can compromise formulation reproducibility [22]. In addition, hydrophobic derivatives such as EC require the use of organic solvents during processing, and more effective strategies are needed to enhance the ocular penetration of larger or structurally complex therapeutic molecules [14]. Addressing these limitations will be essential for advancing the clinical utility of cellulose-based delivery systems.

### 2.2. Protein Based Biopolymers

Protein-based biopolymers, including collagen, gelatin, and silk fibroin, are highly valued in ocular therapeutics due to their exceptional biocompatibility and close structural resemblance to native ocular tissues. These materials provide functional scaffolds that support cellular adhesion and proliferation while enabling sustained drug release. Their tunable degradation profiles and favorable safety characteristics further underscore their suitability for clinical translation (Table 8).

#### 2.2.1. Gelatin

Gelatin, a partially hydrolyzed form of collagen, has emerged as a highly versatile biopolymer for extended ocular drug delivery due to its intrinsic biocompatibility, biodegradability, and mild processing requirements [79]. Its structural similarity to native extracellular matrix components enables excellent ocular tolerance, while its ability to form hydrogels, nanoparticles, microspheres, and adhesive scaffolds provides multiple avenues for sustained and controlled drug release [80,81]. Gelatin is less immunogenic compared to collagen and its precursor and retains informational signals, such as RGD (Arg-Gly-Asp) sequence, thus promoting cell adhesion and proliferation [80]. Gelatin’s physicochemical properties—particularly its abundance of functional groups, tunable crosslinking behavior, and capacity for chemical modification—allow it to encapsulate a wide range of therapeutic agents, including small molecules, peptides, proteins, and nucleic acids [81,82]. These characteristics have positioned gelatin as a promising candidate for addressing the limitations of conventional ophthalmic formulations [19,20,21].

Among gelatin-based systems, gelatin nanoparticles have been the most extensively investigated for ocular applications [83,84]. Their nanoscale dimensions facilitate corneal penetration, while the gelatin matrix serves as a biodegradable reservoir that enables controlled drug diffusion [25,26]. Moreover, surface functionalization with mucoadhesive or targeting ligands has been shown to further prolong precorneal residence time, making gelatin nanoparticles adaptable to a wide range of ophthalmic indications [16,29]. One notable example is the development of gp91 ds-tat (gp91) peptide–encapsulated gelatin nanoparticles (GNP-gp91) for the inhibition of corneal angiogenesis [85]. This formulation exhibited extended corneal retention and effectively suppressed corneal neovascularization with a low dosing frequency, demonstrating strong potential for ophthalmic translation [27]. In another study, mucoadhesive gelatin methacryloyl (GelMA) nanoparticles functionalized with phenylboronic acid (PBA)—termed GelMAP—were engineered for sustained ocular delivery of moxifloxacin (MFX) [86]. The MFX-loaded GelMAP nanosuspension outperformed commercial moxifloxacin eyedrop (Vigamox^®^), showing reduced corneal opacity, lower clinical severity scores, and decreased bacterial burden in infected corneas [86]. Additionally, rosmarinic acid-conjugated gelatin nanogels loaded with diquafosol sodium were developed as a dual-action therapy for dry eye disease, providing both reactive oxygen species (ROS) scavenging and mucin-secreting effects [87]. In vitro and in vivo evaluations demonstrated significant suppression of ROS production, reduced inflammatory mediator expression, and enhanced mucin secretion [28,63].

Gelatin is also frequently used to stabilize and increase the biocompatibility of other nanoparticles [88,89]. For example, silver nanoparticles with primary antimicrobial activity can be combined with gelatin coating, which enhances their performance and safety on the ocular surface [30]. In addition, gelatin improves nanoparticle stability on the cornea, reduces cytotoxicity to surrounding tissues, and increases interaction with corneal structures, which in turn prolongs precorneal retention [89].

Gelatin hydrogels represent another major class of gelatin-based ocular delivery systems, valued for their transparency, tunable mechanical properties, and ability to form in situ under physiological conditions [90,91]. These hydrogels can encapsulate therapeutic agents within a hydrated three-dimensional network, enabling sustained release over periods ranging from hours to several days [59]. Composite hydrogels—such as gelatin–silk fibroin or gelatin–hyaluronic acid systems—exhibit enhanced mechanical stability and slower degradation, making them suitable for chronic ocular diseases that require prolonged therapy [92,93]. In addition to topical applications, injectable gelatin hydrogels have been investigated for intraocular or periocular administration, where they can form localized depots that release drugs gradually while minimizing systemic exposure [94,95].

Beyond nanoparticles and hydrogels, gelatin has also been utilized to fabricate microspheres, nanocomposites, and drug-eluting ocular adhesives [81]. Gelatin microspheres can sustain drug release for days to weeks depending on crosslinking density, offering potential for long-term treatment of inflammatory or infectious ocular conditions [96]. Gelatin-based ocular adhesives and regenerative scaffolds have been developed to simultaneously promote corneal healing and deliver therapeutic agents in a controlled manner [97,98]. These systems are particularly valuable following ocular surgery or trauma, where they can serve as both protective barriers and localized drug reservoirs [97,98]. Electrospun gelatin-based membranes and composite bandages have also shown promise as drug-eluting platforms that support epithelial regeneration while providing sustained therapeutic exposure [99].

#### 2.2.2. Collagen

As the most abundant structural protein in the human body and a major component of the cornea, sclera, and conjunctiva, collagen offers a unique combination of biocompatibility, biodegradability, mechanical tunability, and drug-binding capacity [100]. Collagen’s natural presence in ocular tissues provides an inherent advantage for drug delivery [100,101]. The corneal stroma is composed of approximately 90% type I collagen, arranged in a highly ordered lamellar structure that contributes to transparency and mechanical strength [102,103]. Because collagen is recognized as “self” by the immune system, collagen-based materials typically exhibit minimal inflammatory response, even when applied directly to the ocular surface [104]. This biocompatibility is essential for extended drug delivery systems, which must remain in contact with delicate tissues for prolonged periods.

Another key advantage is collagen’s biodegradability [104]. Enzymes such as collagenases and matrix metalloproteinases, which are naturally present in tears and corneal tissues, can gradually degrade collagen-based matrices. This enzymatic breakdown enables controlled drug release without requiring surgical removal of the delivery system. By modulating parameters such as crosslinking density, collagen concentration, and the incorporation of stabilizing agents, researchers can finely adjust degradation profiles, achieving release durations ranging from several hours to multiple weeks [100].

Collagen hydrogels are among the most widely studied collagen-based ocular delivery systems [100,104,105]. These hydrogels are capable of encapsulating small molecules, peptides, proteins, and even nanoparticles, forming a hydrated three-dimensional network that closely mimics the extracellular matrix. Their high water content promotes comfort upon ocular application, while the intrinsic porosity of the hydrogel structure enables diffusion-controlled drug release.

Collagen corneal shields, originally developed as bandage contact lenses, have been repurposed as platforms for ocular drug delivery [106,107]. BIO-Cor^®^, a commercially available collagen corneal shield produced by Bausch & Lomb Pharmaceuticals (Tampa, FL), is composed of porcine scleral collagen and undergoes complete crosslinking within approximately 72 h after placement on the ocular surface [100]. During dissolution, these shields release incorporated therapeutics in a sustained manner. Compared with conventional eye drops, collagen shields significantly prolong drug residence time by resisting tear dilution and blink-induced clearance, while simultaneously providing a protective barrier that supports epithelial regeneration following surgery or corneal injury [108].

Although beyond the scope of this review, collagen-based implants represent a minimally invasive alternative to repeated intravitreal injections, with mechanical strength tunable through crosslinking or polymer blending to support long-term structural integrity [109,110]. These implants are capable of incorporating a wide range of therapeutics—including corticosteroids, anti-VEGF agents, and neuroprotective peptides—to achieve sustained release in ocular disease models such as glaucoma, diabetic retinopathy, and age-related macular degeneration. Their natural biodegradability contributes to a favorable safety profile, further supporting their potential for chronic ophthalmic use [110,111].

In addition to serving as drug-delivery platforms, collagen itself plays an active role in ocular wound repair. It promotes the migration of macrophages and fibroblasts to the injury site, facilitating the formation of new extracellular matrix [112]. Its high hydrophilicity also makes collagen well suited for ocular wound dressings, enabling the absorption of exudates rich in matrix metalloproteinases and growth factors, which further accelerates tissue regeneration [112,113].

#### 2.2.3. Silk Fibroin

Silk fibroin possesses several intrinsic characteristics that make it highly suitable for ocular applications [114,115,116]. Its excellent biocompatibility has been confirmed in numerous in vitro and in vivo studies, showing minimal inflammation when applied to ocular tissues due to its proteinaceous nature and lack of toxic degradation products [117]. Silk fibroin also offers high optical transparency, essential for corneal or intraocular use, and films, hydrogels, and coatings made from silk can achieve light transmittance comparable to the native cornea [118]. Another major advantage is the tunability of its mechanical and degradation properties [114,119]. By controlling β-sheet content through water annealing, methanol treatment, or physical crosslinking, researchers can adjust stiffness, elasticity, and degradation rates, enabling constructs that remain stable from days to months [117,120,121]. Additionally, silk fibroin can stabilize labile molecules—proteins, peptides, and nucleic acids—through protective hydrogen-bonding networks that reduce denaturation and enzymatic breakdown, a valuable feature for ocular biologics requiring prolonged exposure [115,122].

Silk fibroin hydrogels have been widely explored as systems for extended ocular drug delivery [123,124,125]. Formed through physical crosslinking, enzymatic reactions, or sonication, these hydrogels create a hydrated three-dimensional network capable of encapsulating diverse therapeutics. Their high-water content ensures comfort on the ocular surface, while their porous structure supports diffusion-controlled release [126,127,128]. For instance, silk fibroin hydrogels loaded with cyclosporine A show prolonged release compared with conventional drops [127]. In situ-forming silk hydrogels, which transition from liquid to gel under physiological conditions, further enhance retention and reduce dosing frequency [129].

Silk fibroin films provide another promising platform [19,20,21]. These thin, transparent films can be applied directly to the cornea or used as coatings on contact lenses or implants [24]. Their mechanical strength and flexibility allow them to conform to the ocular surface without irritation [19,22]. Drug-loaded silk fibroin films can release drugs over days to weeks depending on thickness, β-sheet content, and drug–matrix interactions [22,23]. Their transparency ensures they do not interfere with vision, making them suitable for even postoperative care, corneal wound healing, and chronic ocular surface disease management [27,30]. Contact lenses infused or coated with silk fibroin have also been developed to combine prolonged wear with controlled drug release [40].

Silk fibroin microparticles and nanoparticles add further versatility [19,20,21]. Silk fibroin nanoparticles can encapsulate hydrophobic drugs, peptides, or nucleic acids, protecting them from degradation and enabling controlled release, while their size and surface properties can be tuned for enhanced corneal penetration or tissue targeting [16,29]. Microparticles can serve as injectable depots for subconjunctival delivery [27]. Because silk fibroin degrades into non-toxic amino acids, these systems offer a safer alternative to synthetic polymers like PLGA (poly(lactic-co-glycolic acid)), which may generate acidic byproducts [14,22]. Although beyond the scope of this review, studies show that silk fibroin microparticles can sustain the release of anti-VEGF agents, corticosteroids, and neuroprotective compounds, making them attractive for treating posterior segment diseases such as diabetic retinopathy and age-related macular degeneration [27,32,33,34,35].

### 2.3. Synthetic-Natural Hybrid Biopolymers

While natural polymers offer excellent biocompatibility and synthetic polymers provide mechanical strength and tunable degradation, each class has inherent limitations. Hybrid systems that integrate synthetic components such as PEG or PLGA with natural scaffolds like chitosan or collagen have therefore emerged as “smart” delivery platforms. These composites are engineered to enhance mucus penetration, extend precorneal residence, and enable sustained therapeutic release.

#### 2.3.1. PEGylated Biopolymers

Polyethylene glycol (PEG) is a hydrophilic, non-toxic, and highly biocompatible synthetic polymer widely used to modify the properties of natural biopolymers and other synthetic carriers [130,131]. The process of PEGylation—the covalent attachment of PEG chains to a drug or carrier—serves as a “stealth” strategy that protects therapeutic agents from enzymatic degradation and immune clearance, effectively increasing their half-life in the ocular environment [130]. PEGylated systems are particularly effective at overcoming the mucus barrier, as the hydrophilic PEG corona reduces interactions with mucins, allowing for better penetration through the tear film [132].

In ocular drug delivery, PEG is frequently combined with synthetic polymers such as PLGA or PLA to form diblock or triblock copolymers (e.g., PLGA-PEG-PLGA) capable of self-assembling into nanomicelles or forming thermoreversible hydrogels [133,134]. These PEGylated nano-delivery systems typically exhibit reduced surface charge relative to unmodified carriers, which enhances interaction with the ocular surface and promotes deeper tissue penetration [135]. PEGylated PLGA platforms have demonstrated strong therapeutic potential in ocular hypertension. For example, melatonin-loaded PEG-PLGA nanoparticles (MEL-PLGA-PEG NPs) produced a prolonged intraocular pressure–lowering effect compared with conventional formulations [136]. To further improve ocular delivery, two complementary strategies—PEGylation to enhance mucus penetration and cationization via a cationic surfactant to increase cellular uptake—were integrated for cyclosporin A nanosuspension delivery [137]. This dual-modified system achieved superior mucus penetration, enhanced cellular internalization, and extended precorneal retention without inducing ocular irritation [137]. Additionally, PEGylation of natural polymers such as chitosan or albumin has been shown to improve their stability and facilitate the transport of large biomolecules, including bevacizumab, to the posterior segment following topical or subconjunctival administration [16,138].

#### 2.3.2. PLGA–Biopolymer Composites

Poly(lactic-co-glycolic acid) (PLGA) is one of the most widely used synthetic biodegradable polymers in ocular drug delivery owing to its FDA approval and highly tunable degradation kinetics [139,140]. However, PLGA also presents inherent limitations, including the generation of acidic degradation byproducts that may induce local inflammation or destabilize sensitive protein therapeutics [140,141]. To address these challenges, PLGA is frequently incorporated into composite systems with natural biopolymers such as collagen, chitosan, or hyaluronic acid, thereby combining mechanical robustness with improved cellular compatibility [142,143]. A notable strategy involves the development of “Trojan horse” composite systems, in which drug-loaded natural polymer nanoparticles are encapsulated within a larger PLGA microparticle matrix [139,140]. This hybrid configuration mitigates the rapid burst release typically associated with chitosan and enables sustained drug delivery for 120–200 days [141]. Similarly, PLGA/collagen hybrid constructs are being explored as scaffolds for corneal tissue engineering, leveraging the mechanical strength of PLGA alongside the cell-adhesive properties of collagen [142,143]. These composite systems not only prolong drug release but also enhance ocular tolerance by buffering the microenvironment as the synthetic component undergoes degradation [140].

#### 2.3.3. Hybrid Hydrogel Platforms

Hybrid hydrogels represent a significant advancement in “smart” ocular delivery, often utilizing natural polysaccharides (like hyaluronic acid or alginate) crosslinked with synthetic monomers (such as *N*-isopropylacrylamide or PEG) [143,144]. These systems are formulated as liquid eye drops that undergo a sol-to-gel transition in response to physiological temperature, pH, or ionic strength [144]. A notable example is gelatin-g-poly(*N*-isopropylacrylamide), a semisynthetic copolymer that provides sustained pilocarpine release for glaucoma therapy, achieving significant and prolonged intraocular pressure reduction with fewer administrations [144,145].

As discussed earlier, composite microsphere–hydrogel platforms have also been engineered to achieve near zero-order release of high-molecular-weight biologics [143]. In these designs, drug-loaded PLGA or PLA microspheres are dispersed within a thermoresponsive hydrogel such as poly(*N*-isopropylacrylamide) (PNIPAM) [144,146]. Although this strategy has been experimentally applied to the treatment of neovascular diseases in the posterior segment of the eye [147], it also holds considerable potential for treating corneal disorders and glaucoma through ocular surface application.

## 3. Platforms for Extended Ocular Drug Delivery

While biomaterial innovation is indispensable, parallel advances in delivery platforms are equally crucial to ensure their effective deployment on the ocular surface. Advanced delivery platforms are designed to prolong therapeutic effects by overcoming the eye’s rapid clearance mechanisms. Utilizing biopolymer-based systems like in situ hydrogels, nanocarriers, and drug-eluting inserts, these platforms enhance precorneal residence time and bioavailability, providing a more consistent and effective approach to managing complex ocular conditions (Table 9).

### 3.1. In Situ Gelling Systems

In situ gelling systems are one of the advanced drug delivery platforms that exist as low-viscosity liquids before administration and undergo a sol-to-gel transition upon contact with the ocular surface [148]. This phase transformation is triggered by specific physiological stimuli, such as changes in temperature, pH, or ion concentration within the tear fluid [149]. For example, thermosensitive hydrogels like poloxamers remain liquid at room temperature but solidify into a viscoelastic gel at the physiological temperature of the eye [150]. Ion-responsive polymers such as gellan gum (Gelrite^®^) similarly gel upon interacting with tear-film cations like sodium or calcium [151]. These “smart” systems markedly improve ocular bioavailability by extending drug residence time in the conjunctival cul-de-sac [152]. Their mucoadhesive properties help them resist nasolacrimal drainage and blinking-induced clearance [152,153,154]. This extended contact allows for a sustained and controlled release of therapeutic agents, reducing the frequency of administration and improving patient adherence to chronic treatment regimens [152].

Despite these advantages, in situ gels face limitations. Insufficient mechanical strength can lead to premature drug leakage or rapid dissolution, compromising sustained delivery [144]. Some formulations may also cause transient blurred vision or irritation if the gel becomes overly viscous or dense [66]. Current research is therefore focused on developing multi-responsive gels that react to multiple stimuli simultaneously, offering more precise release profiles and improved biocompatibility [144].

### 3.2. Nanoparticles

Nanoparticles (NPs) are colloidal drug carriers typically ranging in size from 10 to 1000 nm, engineered to encapsulate both hydrophilic and lipophilic medications [139,152,155]. They are broadly categorized into nanospheres, where the drug is uniformly dispersed throughout a polymer matrix, and nanocapsules, where the drug is sequestered within a central oily or aqueous core surrounded by a thin polymeric shell [139,155,156]. By utilizing biodegradable and biocompatible polymers such as PLGA, chitosan, and gelatin, these systems protect encapsulated drugs from enzymatic degradation while enabling delivery to specific ocular tissues [139,152,157].

The physical properties of NPs, particularly size and surface charge, are critical factors in overcoming anatomical barriers like the corneal epithelium and the blood-retinal barrier [152,158,159]. Because the cornea and conjunctiva possess a net negative surface charge, cationic nanoparticles (such as those made from chitosan) exhibit superior mucoadhesion through electrostatic interactions, dramatically extending precorneal residence time [158,160,161]. Smaller particles, specifically those under 200 nm [152], are more easily internalized by corneal cells via endocytosis, while hydrophilic particles in the 20–80 nm range may even penetrate the scleral pores to reach the posterior segment [152,159,162,163,164].

NP delivery systems offer the potential to replace traditional eye drops for the management of chronic conditions like glaucoma and dry eye disease [152,153,165]. They provide a sustained release profile that can maintain therapeutic drug levels for several weeks after a single application [166], thereby reducing systemic side effects caused by frequent high-dose drops [139,153,157]. However, challenges remain in the large-scale manufacturing and standardization of NP formulations, particularly regarding particle size homogeneity and long-term stability during storage [140,141,155].

### 3.3. Hydrogels

Hydrogels are three-dimensional, crosslinked polymer networks capable of absorbing and retaining large amounts of water while maintaining structural integrity [167,168,169]. Their high water content and soft, elastic nature closely mimic the eye’s extracellular matrix, contributing to excellent biocompatibility and ocular tolerance [167,169]. They can be fabricated from natural polymers such as collagen, hyaluronic acid (HA), and alginate, or from synthetic polymers like polyethylene glycol (PEG), each offering distinct degradation behaviors and drug-release characteristics [169,170].

In ocular therapy, hydrogels serve as drug depots that can be applied topically to the ocular surface or injected into the vitreous [91,170,171]. Topical hydrogels enhance bioavailability by forming a protective, moisture-retaining layer that gradually releases therapeutic agents, making them well suited for conditions such as dry eye disease and keratitis [170]. Injectable hydrogels for posterior-segment delivery are engineered to release large biologics—such as anti-VEGF proteins—over several months, potentially reducing the need for frequent intravitreal injections in patients with age-related macular degeneration [171,172]. Drug release typically occurs through passive diffusion across the hydrogel’s aqueous pores, which can be tuned by adjusting crosslinking density [173]. A major limitation, however, is the initial burst release, during which a substantial fraction of the drug is rapidly lost [167,171]. To overcome this, composite systems embedding drug-loaded nanoparticles or microparticles within the hydrogel matrix have been developed to achieve more uniform and prolonged release [170,171]. Although these technologies are currently being actively investigated for the treatment of retinal and optic nerve diseases, they also hold strong potential for future application in ocular surface drug delivery. For example, Wang et al. reported an injectable thermosensitive hydrogel drug delivery system engineered to deliver ciliary neurotrophic factor and triamcinolone acetonide for the treatment of traumatic optic neuropathy [174]. They found that retinal ganglion cell survival rate was 31.05 ± 1.41% for the drug-loaded hydrogel system (vs. the control group was 16.79 ± 1.50%) at day 28 [174]. OTX-TKI is a biodegradable, axitinib-loaded hydrogel implant developed by Ocular Therapeutix™. The three-dimensional matrix encapsulates axitinib particles and, once injected, hydrates and gradually dissolves to release the drug over time. The implant is comparable in size to a fine fiber delivered through a 25–27 g needle and is designed to provide sustained release for six months or longer [175]. OTX-TKI is currently being evaluated as an intravitreal therapy for wet AMD, with an ongoing phase III clinical trial (NCT06223958).

### 3.4. Contact Lenses as Drug Reservoirs

Therapeutic contact lenses represent a highly effective non-invasive platform for ocular drug delivery, capable of achieving over 50% bioavailability compared to the 1–5% typically seen with eye drops [176,177,178]. Because the lens sits in close proximity to the cornea, it creates a stagnant thin film of tear fluid that promotes the continuous diffusion of the drug into the eye [177,178]. While traditional “soak and release” methods are simple, they often result in an initial burst release of the drug within the first few hours, failing to provide the long-term therapy required for conditions like glaucoma [178,179].

To extend release durations, advanced strategies such as molecular imprinting and the incorporation of diffusion-modulating barriers like vitamin E can be employed [180,181,182]. Molecular imprinting creates tailored binding sites within the lens matrix that have a high affinity for specific drug molecules, slowing their release [180,181]. Additionally, nanoparticle-laden contact lenses involve dispersing drug-filled nanocarriers within the lens material, creating a double-barrier system that can sustain release for several days or even weeks [182,183,184].

Despite these advancements, the commercialization of drug-eluting contact lenses faces hurdles related to maintaining critical lens properties such as oxygen permeability, optical transparency, and mechanical strength [24,185]. The incorporation of high drug loads or nanoparticles can sometimes cause lens opacification or alter the refractive index, interfering with the patient’s vision [24,186]. Future research aims to optimize these material properties while ensuring that the lenses remain comfortable for extended wear [187].

### 3.5. Ocular Inserts and Films

Ocular inserts and films are solid or semi-solid devices typically composed of biocompatible polymers and placed in the conjunctival fornix [188,189,190]. They are designed to overcome the limitations of eye drops by providing accurate dosing without the need for preservatives [188,189,190]. These platforms are categorized into insoluble inserts, which function via diffusion and must be removed after use, and soluble or bioerodible inserts, which naturally dissolve in the tear fluid, enhancing patient comfort [188,189].

One prominent example of this technology is the collagen shield, which is fabricated from porcine or bovine collagen and gradually dissolves over 12 to 72 h, releasing drugs while simultaneously promoting corneal wound healing [108,188]. Modern advancements include the development of nanofiber-based inserts, produced via electrospinning, which offer a high surface-area-to-volume ratio for rapid yet controlled delivery of large protein drugs or nucleic acids [191,192]. These thin, flexible films settle comfortably on the eye and deliver drugs consistently for days at a time [191,192].

The use of punctal plugs is another specialized form of ocular insert, where a tiny device is placed in the lacrimal punctum or canaliculus to block tear drainage while simultaneously releasing a drug into the precorneal tear film [166,190,193]. While inserts offer superior control over release kinetics, they can occasionally cause discomfort or vision interference [188,189,193]. Current research is focused on optimizing the shape and material friction of these devices to ensure they remain stable in the eye during constant blinking with minimal irritation [166,190,193].

### 3.6. Comparative Evaluation of Ocular Drug Delivery Platforms

Various advanced ocular drug delivery platforms are engineered to overcome ocular drug barriers. In situ gelling systems provide the convenience of liquid instillation with prolonged contact through stimuli-triggered phase transitions, though they often lack mechanical robustness and may cause temporary blurred vision. Nanoparticles protect therapeutic agents and enhance tissue penetration through size and surface-charge effects (e.g., cationic mucoadhesion), but face challenges in large-scale manufacturing and long-term stability. Hydrogels offer a biocompatible, ECM-mimetic reservoir for sustained release, but their clinical utility is frequently limited by an initial burst release. Drug-eluting contact lenses can achieve exceptionally high bioavailability via continuous diffusion, although maintaining optical clarity and oxygen permeability at high drug loads remains difficult. Ocular inserts and films enable precise, preservative-free dosing and support wound healing, but may induce foreign-body sensations or interfere with vision. Microneedle-based systems provide minimally invasive, targeted delivery that bypasses scleral barriers and allows dose-sparing administration, though they require careful optimization to avoid heat- or insertion-related tissue damage. Therefore, selecting an appropriate platform ultimately requires balancing these pharmacokinetic advantages against practical clinical, safety, and manufacturing constraints.

## 4. Strategies for Sustained Ocular Drug Release

**Figure 3:**
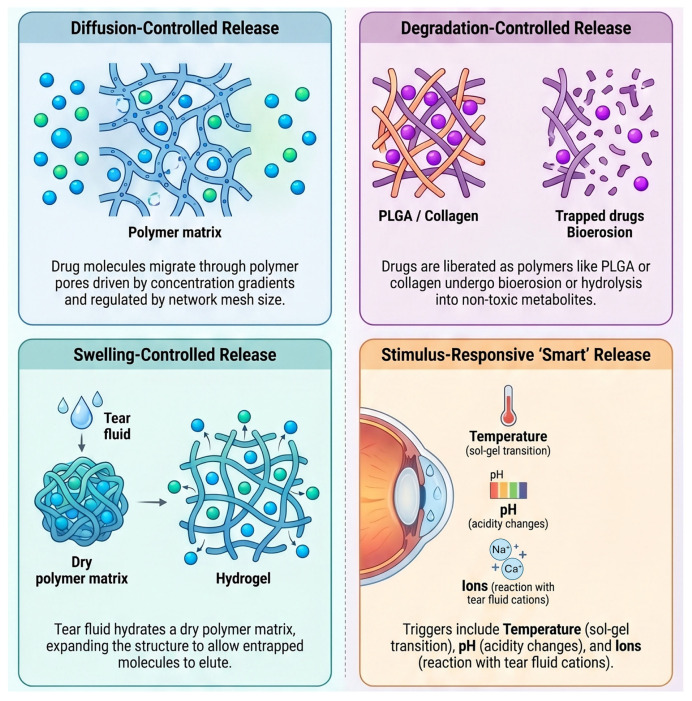
Schematic of the four fundamental drug release mechanisms for ocular delivery via advanced biopolymer systems. Diffusion-controlled release involves drug migration through a crosslinked hydrogel network, with rates regulated by concentration gradients and mesh size. Swelling-controlled release is initiated by the hydration of a dry polymer matrix in tear fluid, expanding pores to facilitate drug elution. Degradation-controlled release synchronizes drug liberation with the bioerosion or hydrolytic cleavage of polymer chains. Stimulus-responsive (triggered) release utilizes platforms that respond to environmental cues, such as temperature-induced sol-gel transitions, pH variations in inflammatory sites, or ion-triggered crosslinking in the tear film. Figure 3 was created using NotebookLM.

Developing effective strategies for sustained release is essential to counteract the eye’s natural clearing processes. By utilizing diverse methodologies such as matrix-controlled diffusion, chemical conjugation, and environmental responsiveness, these systems maintain therapeutic concentrations over extended periods. Such innovations are fundamental to improving clinical outcomes while reducing the dosing frequency (Table 10, Figure 3).

### 4.1. Diffusion-Controlled Release

Passive diffusion serves as the fundamental mechanism for drug delivery from many ocular platforms, relying on the natural migration of drug molecules from a high-concentration region within a carrier to a low-concentration region in the surrounding ocular tissues [145,194,195]. This process is driven entirely by a concentration gradient and does not require external energy [194,195]. The rate of drug diffusion is primarily regulated by the pore size of the delivery system, the crosslinking density of the polymer matrix, and the molecular weight of the therapeutic agent [143,195,196]. In general, small-molecule drugs penetrate these networks more easily than macromolecules, while higher crosslinking density can create smaller pores that increase resistance to drug movement [143,196].

Diffusion-controlled platforms are often categorized into reservoir systems and matrix systems [194,197]. In reservoir systems, such as ocular inserts, a solid drug core is surrounded by a non-biodegradable, semipermeable membrane that acts as a rate-limiting barrier [153,197]. This design allows for more precise control over the release rate, often achieving zero-order kinetics where a constant amount of drug is released over time [141,197]. In matrix systems, the drug is homogeneously dispersed throughout a polymer, and the release rate typically declines as the distance the drug must travel to reach the surface increases [141,197].

Recent clinical advancements have successfully utilized diffusion-controlled mechanisms in sophisticated devices like the Port Delivery System (PDS) with ranibizumab [198,199]. The PDS uses a semipermeable titanium matrix to enable the passive diffusion of large protein molecules from an internal reservoir into the vitreous humor over six months [198,199]. Similarly, therapeutic contact lenses utilize molecular diffusion to release drugs into the post-lens tear film, where the lens protects the medication from rapid clearance by blinking and lacrimal exchange [176,178]. However, a significant challenge for simple diffusion systems is the initial burst release, which can lead to high drug concentrations shortly after administration before stabilizing [141,195].

### 4.2. Swelling-Controlled Release

Swelling-controlled release is a physical strategy where a polymer matrix absorbs biological fluids, such as tears, and undergoes a volume expansion that facilitates the escape of drug molecules [145,170,200]. When a dry or semi-solid hydrogel is introduced into an aqueous environment, it absorbs water, causing the polymeric network to relax and the mesh size to increase [143,200]. This transition from a glassy to a rubbery state allows drugs that were previously trapped or restricted to diffuse out of the expanded structure [143,145]. This mechanism is particularly common in hydrogels and punctual plugs, which are designed to fit or swell to fit anatomical spaces like the punctum [149,166].

The equilibrium swelling ratio, defined as the weight of the fully swollen gel relative to its dry weight, is a critical parameter that determines the rate of network expansion and subsequent drug release [200,201]. By modulating the hydrophilic-hydrophobic composition and the crosslinking density of the polymer, researchers can program release profiles that span from a few hours to several months [143,201]. For example, sodium alginate and certain starch derivatives exhibit high swelling capacities that form a viscoelastic barrier, effectively slowing the clearance of the drug from the ocular surface [202,203].

In addition to regulating drug kinetics, swelling behavior plays a pivotal role in patient comfort and visual performance [66,187]. In applications like therapeutic contact lenses or ocular dressings, excessive swelling can lead to lens displacement, visual blurring, or a feeling of tightness in the eye [187,204]. Conversely, insufficient swelling may compromise the device’s ability to provide adequate lubrication and moisture retention. Therefore, ophthalmic swelling-controlled systems must be carefully designed to maintain a moderate and stable swelling state under physiological conditions to ensure both therapeutic efficacy and patient tolerance [66,201].

### 4.3. Degradation-Controlled Release

Degradation-controlled release relies on the chemical breakdown of a polymer matrix into smaller, biocompatible fragments, which triggers the liberation of the encapsulated drug [140,205,206]. This process typically occurs through nonenzymatic hydrolysis of ester linkages or enzymatic reactions that cleave the polymer backbone [140,206]. A major advantage of this strategy is that the delivery vehicle eventually dissolves and is metabolized by the body, eliminating the need for surgical removal after the drug load is depleted [205,206]. Common materials used for these systems include PLGA (poly lactic-co-glycolic acid), PLA (polylactic acid), and chitosan [139,140].

The kinetics of degradation can be categorized into bulk erosion and surface erosion [161,207]. In bulk erosion, which is typical for PLGA, water penetrates the entire matrix, leading to a random cleavage of polymer chains and a release profile that is often multi-phasic [205,207]. In contrast, surface erosion occurs when the polymer degrades only at the exterior interface, allowing for a consistent, zero-order release rate that is proportional to the surface area of the device [207,208]. Polyanhydrides are frequently cited as excellent examples of surface-eroding materials due to their high aqueous reactivity and predictable degradation patterns [208,209].

Clinical implementation of degradation-controlled release is seen in FDA-approved implants like Ozurdex^®^ (dexamethasone intravitreal implant) and Durysta^®^ (bimatoprost intracameral implant) [210,211]. Ozurdex^®^ utilizes a PLGA-based matrix to release dexamethasone over six months as the polymer gradually hydrolyzes into lactic and glycolic acids [210,212]. However, the use of these materials can sometimes create an acidic microenvironment during degradation, which may potentially cause local inflammation if not properly managed [139,141]. Ongoing research focuses on tuning the degradation duration relative to the treatment duration to prevent the buildup of multiple undegraded implants in the eye [205,206].

### 4.4. Stimulus-Responsive Release

Stimulus-responsive systems are advanced platforms that undergo a dramatic change in their physical state in response to specific environmental triggers [149,213,214]. These triggers can be physical (temperature, light, ultrasound) or chemical (pH, ionic strength, redox state) [149,213]. The most common application in ophthalmology is the sol-to-gel transition, where a low-viscosity liquid is instilled as an eye drop and rapidly transforms into a viscoelastic gel upon contact with the ocular surface [66,214,215]. This transformation creates a bio-adhesive drug depot that resists nasolacrimal drainage and prolongs the residence time of the medication [66,215].

Thermoresponsive hydrogels, such as those based on poloxamers or poly(*N*-isopropylacrylamide), are designed to be liquid at room temperature (20–25 °C) but jellify at the physiological temperature of the eye (35–37 °C) [149,216]. pH-responsive hydrogels utilize ionizable functional groups like carboxyl or amino groups that change their degree of ionization when they encounter the mildly basic environment of the tear film (pH ~ 7.8) [213,216]. Furthermore, ion-responsive systems like gellan gum (Gelrite^®^) react with the monovalent and divalent cations (such as Na^+^ and Ca^2+^) naturally present in tears to form a crosslinked, stable matrix [215,217].

Ultrasound-responsive hydrogels can be engineered by loading polymeric hydrogels or nanocarriers—such as nanobubbles—with therapeutic agents and subsequently exposing them to ultrasound after administration [218,219]. The applied ultrasound induces cavitation and localized temperature elevation, which in turn leads to the rupture of the polymeric chains or the collapse of the nanobubble structure, thereby triggering drug release. This delivery strategy has demonstrated advantages in enhancing drug penetration across various ocular barriers, including the cornea [219]. However, concerns remain regarding the ultrasound-induced temperature increase, as excessive heat may pose a risk of damage to the eye’s sensitive tissues.

The next generation of these technologies focuses on multi-responsive gels that can sense and respond to several stimuli simultaneously or sequentially [144,149,213]. For instance, a gel might respond to both temperature and specific disease biomarkers, such as overexpressed enzymes at an inflammatory site, to achieve on-demand, targeted drug release [144,148]. This approach allows for spatiotemporal control over drug delivery, precisely matching the release profile to the progression of a patient’s unique pathological condition while minimizing systemic exposure and side effects [144,148,149].

### 4.5. Mucoadhesive with Retention Time

Mucoadhesion is the ability of certain materials to adhere to the mucosal membranes of the eye, providing a temporary but significant retention time for the delivery system [66,152,220]. This strategy is designed to overcome the rapid clearance caused by reflex blinking and tear turnover [66,152,153]. Mucoadhesive polymers function through several mechanisms, including electrostatic interactions, hydrogen bonding, and physical interpenetration of polymer chains into the viscoelastic mucus gel [66,220]. By binding to the mucin layer, these platforms can extend the contact time from minutes to several hours or even days [66,152].

Chitosan is one of the most widely researched mucoadhesive polymers due to its polycationic nature [160,221]. Since the cornea and conjunctiva possess a net negative surface charge, positively charged chitosan particles bind effectively through electrostatic attraction [152,160,161]. Chitosan also has the unique ability to transiently open tight junctions between epithelial cells, which further enhances the penetration of drugs through the paracellular pathway [160,222]. Other natural polymers like Hyaluronic Acid (HA) also exhibit strong mucoadhesion; HA acid groups interact with the sialic acid portion of eye mucin via non-covalent bonds, achieving superior retention compared to many other biopolymers [152,223].

Beyond simple physical adhesion, some mucoadhesive polymers like HA can provide a receptor-mediated targeting effect by binding to CD44 on the surface of corneal epithelial cells [223,224]. This interaction not only secures the drug delivery system in place but can also promote corneal wound healing and provide synergistic therapeutic benefits [223,224]. By incorporating these materials as coatings for nanoparticles or as viscosity-enhancing agents in viscous solutions, researchers have successfully increased the ocular bioavailability of drugs like timolol and dexamethasone by factors of two to eight-fold compared to conventional formulations [152,160,225].

### 4.6. Comparative Evaluation of Release Mechanisms and Retention Enhancing Strategies

To address the mechanistic complexity of sustained ocular drug delivery, it is essential to critically compare major release strategies—diffusion, swelling, degradation, stimuli-responsiveness, and mucoadhesion—based on their underlying physical and chemical drivers. Diffusion-controlled systems rely on concentration gradients; reservoir-type designs can achieve near zero-order kinetics, whereas matrix systems often exhibit an initial burst release as drug molecules migrate toward the surface. Swelling-controlled platforms use water-induced polymer relaxation to expand the network mesh, a feature advantageous for anatomically constrained devices such as punctal plugs, though excessive swelling in contact lenses may compromise optical clarity and lens stability. Degradation-mediated release provides the benefit of biological elimination without the need for device retrieval; however, bulk-eroding polymers such as PLGA generate acidic byproducts that may provoke local inflammation. Stimuli-responsive systems enable convenient administration through in situ phase transitions triggered by tear-film pH, temperature, or ionic composition, yet these materials often lack sufficient mechanical robustness to withstand blinking-induced shear forces. Mucoadhesive strategies enhance retention by forming electrostatic, hydrogen-bonding, or interpenetrating interactions with the ocular mucin layer, extending precorneal residence from minutes to hours or even days and counteracting rapid clearance by blinking and nasolacrimal drainage.

Selecting an optimal release mechanism therefore requires balancing the desired pharmacokinetic profile with clinical constraints. Diffusion- and swelling-based systems offer predictable physical control, whereas degradation-mediated, stimuli-responsive, and mucoadhesive platforms provide more biologically integrated solutions that reduce procedural burden and maximize therapeutic residence time.

## 5. Applications of Biopolymer-Based Systems in Ocular Therapeutics

A wide range of biopolymers has been actively investigated and developed for ocular drug-delivery applications. Table 11 provides a comprehensive overview of representative experimental studies utilizing various biopolymers to achieve extended ocular drug delivery. These studies highlight the versatility of materials such as hyaluronic acid (HA), chitosan, alginate, and HPMC in formulating diverse platforms, including ocular inserts, films, implants, and microneedle systems. By incorporating therapeutic agents ranging from anti-glaucoma drugs like timolol to immunomodulators such as cyclosporine A, these biopolymer-based systems have demonstrated superior performance in maintaining therapeutic drug levels and enhancing bioavailability compared with conventional topical administration.

## 6. Recent Advances and Future Directions

Recent advances in this field have accelerated, leading to the development of numerous technologies and products that extend beyond laboratory-level research toward real clinical application. Several biopolymer-based platforms for ocular surface drug delivery are also undergoing ongoing clinical evaluation (Table 12).

### 6.1. Advances in Topically Applied Nanocarriers

Nanotechnology has emerged as a cornerstone in the development of next-generation ocular drug delivery systems [152,158,159]. By utilizing carriers in the 10–1000 nm range, the solubility of hydrophobic drugs can be enhanced and their residence time on the ocular surface [158,159,162]. Nanomicelles are particularly promising for solubilizing lipophilic agents in aqueous solutions [158,242]. For instance, Cequa^®^, an FDA-approved 0.09% cyclosporine-A nanomicellar solution, offers significantly improved efficacy and better biocompatibility for dry eye disease compared to traditional oil-based formulations [242,243]. Similarly, polymeric and lipid nanoparticles protect encapsulated drugs from enzymatic degradation while enabling controlled release [139,158,159]. Cationic nanocarriers are especially effective for anterior segment delivery because their positive surface charge interacts electrostatically with the negatively charged mucin layer of the cornea, dramatically extending residence time [158,160,161].

Another major advancement is the development of “smart” nanoparticle loaded in situ gelling systems [66,149]. These formulations are instilled as low-viscosity liquids and undergo a sol-to-gel transition triggered by physiological stimuli such as temperature, pH, or ionic strength in the tear fluid [149,215]. These bioadhesive networks bind to the ocular surface, providing a sustained-release depot that reduces dosing frequency and improves bioavailability for medications like timolol and moxifloxacin [66,215]. Abou-Taleb et al. combined Pluronic F-127, a thermosensitive polymer, with pH-sensitive, permeability-enhancing chitosan as a gelling agent to deliver propamidine-isethionate [244]. In-vitro pharmaceutical and antiprotozoal studies against Acanthamoeba keratitis showed that the propamidine-isethionate -chitosan nanoparticle in-situ gel had smaller particle size, higher zeta potential, sustained 24-h drug release, and greater amoebic inhibition after 24 h compared with other formulations [244]. Kalaria et al. developed gemifloxacin mesylate nanoparticles via ionic gelation with sodium tripolyphosphate crosslinking chitosan and showed biphasic release with 15% released in 1 h and 90.53% over 24 h [245]. Incorporation into a Poloxamer-407 in situ gel produced sustained release and strong antimicrobial activity against Gram-positive and Gram-negative bacteria [245].

### 6.2. Advances in Sustained-Release Implants and Devices

Although these technologies are not designed for ocular surface drug delivery, the field of intraocular implants for retinal disease treatment has recently become one of the most actively investigated areas. The technologies being developed in this area can be adapted, with minor modifications, for application to chronic ocular surface diseases, and may also be utilized in intrascleral or intraocular implant approaches for treating anterior-segment disorders such as glaucoma. For chronic vitreoretinal diseases such as age-related macular degeneration and diabetic retinopathy, intravitreal injections are the standard route but carry risks of endophthalmitis and retinal detachment [246,247,248]. Intraocular implants have been engineered to replace these frequent injections by providing localized drug release for months or years [247,248,249]. These are categorized as biodegradable or non-biodegradable [248,249]. As discussed previously, Ozurdex^®^ is a prominent example of a biodegradable PLGA-based implant that releases dexamethasone for up to six months before dissolving completely [210,212]. In contrast, non-biodegradable implants like Retisert^®^ can deliver fluocinolone acetonide for up to three years, though they require surgical removal or replacement [250,251].

A transformative shift in posterior segment therapy is the port delivery system with ranibizumab (Susvimo™) [198,252]. This refillable, non-biodegradable implant is surgically placed in the eye and allows for the continuous release of monoclonal antibodies over six months, significantly reducing the burden of monthly intravitreal injections for patients with wet age-related macular degeneration [198,252].

### 6.3. Innovative Platforms: Contact Lenses and Microneedles

Therapeutic contact lenses are being reimagined as drug delivery vehicles that can provide up to 50% more bioavailability compared to eye drops [176,178,179]. Modern techniques such as molecular imprinting and the incorporation of nanoparticle-laden matrices have addressed the limitations of simple “soak and release” methods, which often suffered from rapid burst release [179,184,253]. These engineered contact lenses can now deliver anti-glaucoma or anti-inflammatory drugs for several weeks while maintaining the optical transparency required for vision [178,179,184].

Furthermore, microneedle technology offers a minimally invasive alternative for targeting the suprachoroidal space [254,255]. Tiny needles, often less than 1 mm in length, can bypass the scleral barrier with high precision to deliver drugs directly to the posterior tissues [254]. Dissolvable polymeric microneedle patches have demonstrated the ability to form drug depots within ocular tissues, providing sustained release of drugs like pilocarpine without the pain of standard needles [254,256]. Kim et al. evaluated hollow microneedle delivery of antiglaucoma drugs to the supraciliary space and found a strong, dose-dependent IOP reduction in rabbits, achieving nearly a 100-fold dose-sparing effect compared with topical eyedrops [257]. This targeted approach suggested improved safety and long-lasting therapeutic action from a single injection. Roy et al. developed dissolving PVA–PVP microneedles shaped like contact lenses for transcorneal pilocarpine delivery [258]. Ex vivo porcine-eye studies showed faster administration and markedly higher pilocarpine flux and aqueous-humor availability compared with eye drops. Bhatnagar et al. developed PVA–PVP polymeric microneedle arrays loaded with besifloxacin to improve transcorneal delivery for ocular infections. The microneedles penetrated about 200 µm into the cornea and fully dissolved within 5 min, significantly increasing besifloxacin deposition and permeation [259].

### 6.4. Future Directions and Clinical Challenges

**Figure 4:**
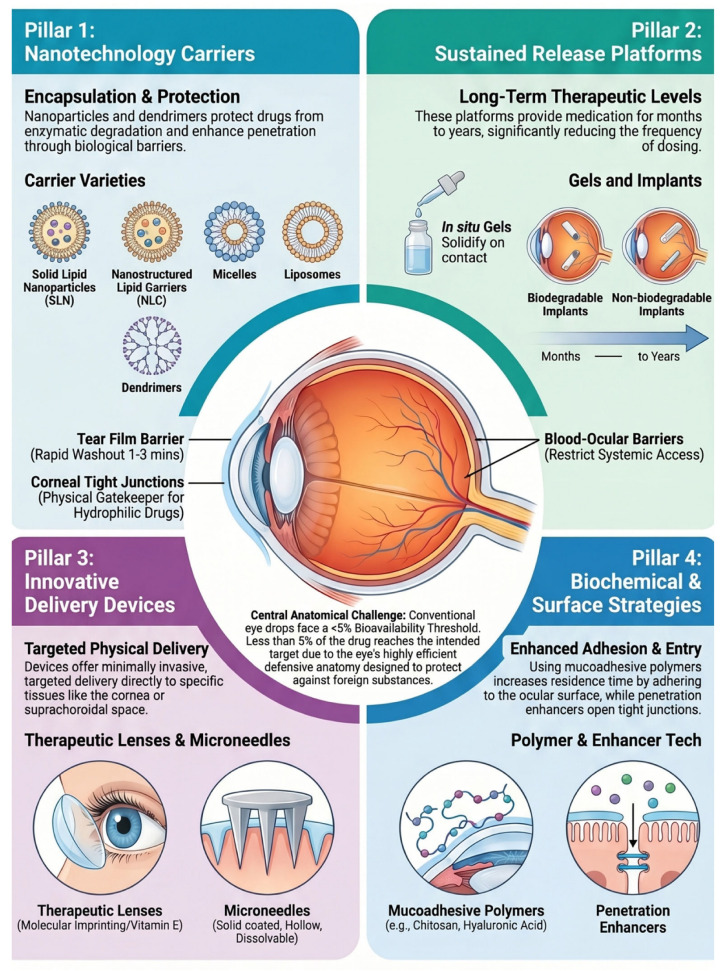
Contemporary Strategies for Overcoming Ocular Anatomical Barriers. Conventional topical delivery suffers from low bioavailability due to the tear film barrier, corneal tight junctions, and blood-ocular barriers. To address these challenges, four distinct delivery modalities are employed: Nanotechnology Carriers utilize lipid-based (SLN, NLC, liposomes) and polymeric (micelles, dendrimers) systems to protect cargo and enhance penetration; Sustained Release Platforms, including in situ hydrogels and intraocular implants, provide long-term therapeutic levels ranging from days to years; Innovative Delivery Devices, such as therapeutic contact lenses (molecular imprinting and vitamin E barrier) and microneedles, allow for targeted physical delivery through various administration routes; and Biochemical Strategies leverage mucoadhesive polymers and penetration enhancers to increase residence time and temporarily modulate epithelial permeability. Together, these integrated approaches aim to minimize systemic side effects while maximizing localized therapeutic efficacy through controlled, site-specific drug release. Created using NotebookLM.

To date, extensive research and development efforts have led to the establishment of multiple strategies designed to enhance the efficiency of ocular drug delivery. These approaches are either currently employed in clinical practice or have attained a degree of technological maturity that positions them on the verge of clinical translation (Figure 4).

Future research in ocular drug delivery is increasingly focused on the evolution of next-generation biopolymer derivatives. Specifically, advancing the chemical profiles of chitosan, hyaluronic acid (HA), alginate, and cellulose is essential to achieve improved solubility, enhanced mucoadhesion, and tunable degradation rates [22,23,260]. By optimizing these physicochemical properties, researchers aim to extend precorneal residence time and ensure a more controlled, sustained therapeutic release, thereby overcoming the inherent physiological barriers of the eye.

A primary frontier in this field is the development of multifunctional, stimuli-responsive platforms. These “smart” delivery systems, such as in situ forming hydrogels or nano-composites, are designed to release pharmacological agents on-demand in response to specific environmental triggers, including pH, temperature, light, or enzymatic activity [23,154,236]. For instance, pH or temperature-sensitive cellulose and HA hydrogels represent a significant avenue for precision ocular therapy. Such advancements are particularly promising not only for anterior segment disease but also for treating posterior segment diseases, as HA-based microneedles and injectable hydrogels could substantially reduce the frequency and discomfort of intravitreal injections [27].

Technological integration also plays a crucial role in the next generation of ocular therapeutics. The convergence of biopolymers with smart contact lenses, biosensing technologies, and wearable devices allows for a dual-functional approach: simultaneous drug delivery and real-time monitoring of ocular biomarkers [40,260]. Furthermore, the application of 3D printing provides the capability to manufacture customized ocular implants tailored to a patient’s unique anatomical dimensions, enhancing both comfort and efficacy [213,261].

The field is also shifting toward bioinspired materials and innovative molecular strategies. One such approach involves melanin-binding peptides that utilize the eye’s intrinsic pigments as a sustained-release platform, effectively creating polymer-free drug depots [144,261,262]. Additionally, hybrid systems—such as alginate-chitosan or alginate-PLGA composites—are being developed to leverage the synergistic effects of multiple materials to enhance mechanical strength and mucoadhesion.

Despite these scientific milestones, the transition from laboratory research to clinical application faces significant manufacturing and regulatory hurdles [139,140]. While biopolymer-based extended ocular drug delivery systems have produced elegant experimental results, a substantial translational gap remains between laboratory success and clinical implementation. To advance these technologies toward widespread clinical use, several practical barriers must be addressed, spanning patient-centered physiological considerations as well as regulatory and manufacturing challenges.

A primary concern for any topically applied system is its impact on visual function and patient comfort. High-viscosity formulations, such as those incorporating cellulose derivatives, can prolong ocular residence time but may cause temporary blurred vision, eyelid crusting, and general discomfort. For drug-eluting contact lenses, maintaining optical transparency and oxygen permeability is essential; high drug loading or nanoparticle incorporation can alter refractive properties or induce lens opacification, compromising vision. Similarly, the physical presence of ocular inserts or large hydrogel depots may produce foreign-body sensations, reducing patient adherence. Future designs must therefore prioritize biomaterials that provide adequate lubrication and mimic the native extracellular matrix to ensure long-term tolerability.

Stability, sterility, and shelf-life also represent critical hurdles in the transition to commercial products [140,145]. Many biopolymers are sensitive to conventional sterilization methods—heat or gamma irradiation can induce premature degradation or crosslinking, altering release kinetics and mechanical integrity [263]. Ensuring long-term stability of hydrogels and biologically conjugated systems is equally important to prevent drug leakage or polymer breakdown during storage. Natural biopolymers such as hyaluronic acid and collagen further introduce batch-to-batch variability in molecular weight and purity, complicating efforts to establish consistent shelf-life standards [264].

Scalability remains a major obstacle for nanocarriers and complex hybrid systems. Although nanoparticles can be produced reliably at the laboratory scale, achieving uniform particle size and reproducibility in large-scale manufacturing is challenging [139,140]. Advanced fabrication techniques—such as molecular imprinting for contact lenses or 3D bioprinting for customized implants—often entail high production costs, potentially limiting accessibility compared with conventional eye drops. Cost-effective, quality-controlled manufacturing processes are therefore essential for clinical adoption.

Regulatory classification presents an additional barrier, as many extended-release systems fall at the interface of “drug” and “device,” resulting in complex approval pathways. Regulatory agencies require comprehensive safety data, including the biocompatibility of degradation products. For example, although PLGA is FDA-approved, its acidic byproducts may induce local inflammation, necessitating careful monitoring during repeated dosing [139,140]. Natural proteins such as silk fibroin degrade into non-toxic amino acids but raise concerns regarding immunogenicity and source traceability [117]. Clinical trials must demonstrate not only efficacy but also that long-term or repeated use does not lead to accumulation of undegraded material that could affect ocular anatomy or intraocular pressure.

Ultimately, the clinical success of these advanced platforms depends on their ability to provide clear therapeutic advantages that justify increased cost and complexity [22,23]. Future research should prioritize “smart” systems capable of on-demand release in response to disease-specific biomarkers, enabling personalized therapy and further reducing dosing frequency. However, until challenges related to manufacturing scalability, long-term safety, and regulatory harmonization are resolved, many of these sophisticated experimental systems will remain on the threshold of translation. Addressing these practical constraints through interdisciplinary collaboration among material scientists, pharmacists, and clinicians will be essential to advance the next generation of ophthalmic drug delivery technologies.

## 7. Conclusions

Biopolymer-based extended drug-delivery systems represent a major advancement in overcoming the physiological barriers of the eye. By harnessing the unique physicochemical properties of materials such as chitosan, hyaluronic acid, and silk fibroin, researchers have developed platforms that markedly enhance drug bioavailability and patient adherence. The progression from simple eye drops to sophisticated modalities—including stimuli-responsive hydrogels, mucoadhesive nanocarriers, and drug-eluting contact lenses—has enabled more precise and sustained therapeutic delivery for both chronic and acute ocular diseases. These technologies not only prolong drug action but also reduce systemic exposure and decrease dosing frequency.

Despite these advances, translating laboratory innovations into clinical practice remains challenging. Issues related to manufacturing scalability, long-term safety, and regulatory approval must be addressed before widespread clinical adoption can occur. Future research should prioritize the development of “smart” delivery systems capable of real-time monitoring and on-demand release, thereby further personalizing ocular therapy. Ultimately, the integration of material science with clinical ophthalmology has the potential to transform the standard of care, offering more effective, comfortable, and reliable treatments for diseases of both the ocular surface and the posterior segment.

## Figures and Tables

**Table 1:** Representative Biopolymer-Based Ocular Surface Drug-Delivery Platforms Commercialized Since 2000.

Product Name	Active Pharmaceutical Ingredient	Biopolymer Used	Formulation Type	Target Ocular Surface Disease/Indication	Approval Year
Mikelan LA^®^ (Carteolol LA)	Carteolol Hydrochloride (1%/2%)	Alginic Acid	Ion-activated in situ gelling solution	Glaucoma, Ocular hypertension	2007
AzaSite^®^	Azithromycin (1%)	Polycarbophil (DuraSite^®^ Mucoadhesive Platform)	Synthetic cross-linked polymeric gel-forming suspension	Bacterial conjunctivitis & blepharitis	2007
Akten^®^	Lidocaine Hydrochloride (3.5%)	Hydroxypropyl Methylcellulose	High-viscosity mucoadhesive surface gel	Preoperative local anesthesia	2008
Besivance^®^	Besifloxacin (0.6%)	Polycarbophil (DuraSite^®^ Mucoadhesive Platform)	Synthetic cross-linked polymeric suspension	Bacterial keratitis & conjunctivitis treatment	2009
Zirgan^®^ (Virgan^®^ in EU)	Ganciclovir (0.15%)	Carbomer 974P (Polyacrylic acid hydrogel)	Mucoadhesive topical ophthalmic gel	Herpetic keratitis (Dendritic corneal ulcers)	2009
Moxeza^®^ (also sold as Optimox^®^ XG)	Moxifloxacin Hydrochloride (0.5%)	Xanthan Gum	In situ viscous eye drops (Mucoadhesive solution)	Bacterial keratitis & conjunctivitis treatment/prophylaxis	2010
Tobradex^®^ ST	Tobramycin (0.3%) & Dexamethasone (0.05%)	Xanthan Gum (DuraSite^®^ customized matrix)	Mucoadhesive suspension platform	Postoperative ocular inflammation with bacterial infection risk	2011
Ilevro^®^	Nepafenac (0.3%)	Guar Gum & Carboxymethylcellulose	Mucoadhesive viscous suspension	Postoperative pain & inflammation associated with cataract surgery	2012
Dextenza^®^	Dexamethasone (0.4 mg)	Poly(ethylene glycol)-based bioresorbable matrix	Intracanalicular hydrogel insert (Sustained surface elution)	Postoperative inflammation, pain, and ocular surface flare-ups	2018
Inveltys^®^	Loteprednol Etabonate (1.0%)	Poloxamer 407 (Pluronic F127 block copolymer)	Mucus-Penetrating Nanoparticles (MPP) suspension	Postoperative inflammation and pain following ocular surgery	2018
Cequa^®^	Cyclosporine A (0.09%)	Polyoxyl 40 hydrogenated castor oil & Octoxynol-40 (NCELL^®^ Polymeric Nanomicellar Technology)	Aqueous, preservative-free polymeric nanomicellar solution	Dry Eye Disease & Ocular surface chronic inflammation	2018
Eysuvis^®^	Loteprednol Etabonate (0.25%)	Poloxamer 407	Mucus-Penetrating Nanoparticles (MPP) suspension	Short-term treatment of Dry Eye Disease	2020
Acuvue^®^ Theravision™	Ketotifen Coumarate (0.019 mg)	Etafilcon A cross-linked hydrogel matrix	Drug-eluting hydrogel contact lens (Continuous surface elution)	Allergic conjunctivitis & ocular surface itching	2022
Xdemvy^®^	Lotilaner (0.25%)	Hydroxypropyl Methylcellulose with castor oil vehicle	Viscous targeted ectoparasiticide solution	Demodex blepharitis	2023

**Table 2:** Advantages and Disadvantages of Polysaccharide Biopolymers as Ocular Surface Drug Delivery Platforms.

Biopolymer	Advantages	Disadvantages
Hyaluronic Acid	Outstanding biocompatibility and natural eye lubrication. Active mucoadhesion via specific binding to CD44 receptors on the ocular surface. Promotes corneal epithelial wound healing.	Rapid enzymatic degradation by native hyaluronidase, leading to short drug-release windows. Weak mechanical stability when uncrosslinked. Relatively high production cost.
Chitosan	Strong electrostatic mucoadhesion because its positive charge binds tightly to negatively charged mucin. Enhances drug permeability by transiently opening tight junctions in the corneal epithelium Inherent antimicrobial properties.	Poor solubility at physiological pH (7.4), often requiring slightly acidic formulations that can sting or irritate the eye. Potential cell toxicity at higher concentrations or high degrees of deacetylation.
Alginate	Excellent in situ gelling capability; it transitions from a liquid drop to a firm gel upon contact with calcium ions in natural tears. Highly cost-effective, abundant, and structurally stable. Low toxicity and high patient comfort.	Highly porous gel networks often lead to an unwanted “burst release” of hydrophilic drugs. Potential immunogenicity if the polymer contains trace impurities from its original algal source.
Cellulose Derivatives	Exceptional passive viscosity-building properties that prolong precorneal residence time. Highly stable, chemically inert, and universally accepted by regulatory bodies (FDA approved). Very affordable and accessible.	Lacks specific biological or ionic interactions (relies purely on mechanical thickness). Higher concentrations cause temporary blurred vision, crusting around the eyelids, and patient discomfort.

**Table 7:** Cellulose-based biopolymers for ocular drug delivery.

Material	Key Properties	Drug Delivery Characteristics	Applications
Cellulose	Insoluble, highly crystalline structure	Limited use due to poor solubility	Rarely used directly
HPMC	Water-soluble, viscosity-enhancing	Reduces tear drainage, prolongs residence time	Eye drops, in situ gels
CMC	Anionic, hydrophilic	Improves lubrication and retention	Artificial tears
MC	Thermoresponsive behavior	Contributes to sol-gel transition	In situ gel systems
HEC	Hydrophilic, viscosity-modifying	Enhances formulation stability and retention	Ophthalmic formulations

**Table 8:** Comparison of protein-based biopolymers.

Material	Key Properties	Drug Delivery Characteristics	Applications
Gelatin	Derived from collagen, contains RGD motifs, low immunogenicity	Promotes cell adhesion, forms hydrogels and nanoparticles	Drug carriers, ocular adhesives
Collagen	Major corneal ECM component, highly biocompatible	Enzymatic degradation enables controlled release	Corneal implants, wound healing
Silk fibroin	Transparent, tunable β-sheet structure, high mechanical strength	Sustained drug release, stabilizes biologics	Films, contact lenses, nanoparticles

**Table 9:** Comparison of ocular drug delivery platforms.

Platform	Description	Key Features
Nanoparticles	Colloidal carriers (10–1000 nm)	Protect drugs, enable controlled release
Hydrogels	Crosslinked polymer networks	Sustain drug release, mimic ECM
In situ gels	Liquid-to-gel transition systems	Triggered by physiological stimuli
Contact lenses	Drug reservoirs on cornea	Continuous drug diffusion
Ocular inserts	Solid/semi-solid devices	Precise dosing, prolonged release

**Table 10:** Drug release mechanisms in ocular delivery system.

Mechanism	Principle	Key Controlling Factors	Characteristics
Diffusion-controlled	Concentration gradient-driven release	Pore size, polymer density	Gradual release
Swelling-controlled	Water uptake and polymer expansion	Swelling ratio, hydrophilicity	Increased mesh size
Degradation-controlled	Polymer breakdown	Degradation rate	Sustained release
Stimulus-responsive	Environmental trigger response	Temperature, pH, ions	On-demand release
Mucoadhesive	Interaction with mucin layer	Charge, bonding interaction	Prolonged retention

**Table 11:** Studies Developing Ocular Surface Drug Delivery Systems Using Biopolymers.

Drug	Biopolymers Used	Insert/Film/Implant/Microneedle	Administration Route	Target Disease	Performance and Results	References
Timolol maleate	HA & Itaconic acid	Crosslinked film	Topical	Glaucoma	Continuously reduced IOP in rabbits for more than 10 h.	[226]
Cyclosporine A	HA	Ophthalmic insert	Topical	Dry eye	Promoted drug accumulation in the sclera; release rates precisely modulated by polymer ratios.	[227]
Levofloxacin	HPMC, Sodium alginate, and Gelatin	Film insert	Topical	Infection	Excellent mechanical strength and uniform distribution with biphasic release.	[228]
Brinzolamide	HPMC	Sustained-release insert (MeltSerts)	Topical	Glaucoma	Controlled drug release (69% at 8 h) and non-irritating for glaucoma management.	[229]
Moxifloxacin	Sodium hyaluronate	Liposomal moxifloxacin insert	Topical	Infection	71.2% release at 30 min with zero-order kinetics.	[230]
Ciprofloxacin HCl	Hydroxypropyl cellulose (HPC)	3D-printed insert	Topical	Infection	Sustained drug release for 24 h; outperformed marketed eye drops.	[231]
Tobramycin	HA methacrylate (HAMA)	3D-printed contact lens-like patch	Topical	Infection	Sustained drug release and effective inhibition of *Pseudomonas aeruginosa* biofilms.	[232]
Indomethacin	Gellan gum	Scleral implant	Scleral	Keratitis/uveitis	Facilitated sustained release and prolonged therapeutic effect.	[233]
Bevacizumab	Chitosan	Ocular implant	Subconjunctival	Neovascularization	Intended for CNV treatment; improved drug retention.	[234]
Moxifloxacin	HA	Intraocular membrane implant	Anterior chamber	Infection	Sustained release of the antibiotic for 5 days.	[235]
Ranibizumab	Collagen & PEGDM	Sheet-type device (implant)	Scleral	Neovascularization	Continuously delivered drug for 18 weeks and significantly suppressed CNV.	[236]
Curcumin	HA	Dissolvable microneedle patch	Topical	Inflammation (uveitis, infection)	Sustained release over 8 h and enhanced pre-corneal retention to 3.5 h.	[237]
Anti-angiogenic monoclonal antibody (DC101)	HA & Methacrylated HA (MeHA)	Double-layered microneedle patch	Topical	Neovascularization	Microneedles separated and remained in tissue, maintaining sustained release over 3 days.	[238]
Sodium fluorescein, Amphotericin, and FITC-dextran	PVP (various molecular weights)	Dissolving microneedle	Topical (Cornea and Sclera)	Keratitis	demonstrated high mechanical strength; 10-fold increase in macromolecule delivery compared to topical administration.	[239]
Dexamethasone (in nanomicelles)	PNIPAAm51-b-PGA10	Dissolving microneedle	Intrascleral	Inflammation	Increased polymer content (30%) reduced height reduction to 25% during compression; successfully delivered in situ forming nanomicelles.	[240]
Amphotericin B (in liposomes)	PVP and PVA	Dissolving microneedle	Topical (Cornea)	Keratitis	Significantly reduced *Candida albicans* load in ex vivo and rabbit models; effectively treated fungal keratitis	[241]

HA: hyaluronic acid, HPMC: hydroxypropyl methylcellulose, IOP: intraocular pressure, CNV: choroidal neovascularization.

**Table 12:** Investigational Biopolymer Platforms for Active Drug Delivery (ClinicalTrials.gov, Post-2020).

Candidate/Platform	Active Pharmaceutical Ingredient (API)	Biopolymer/Polymeric Matrix Used	Formulation Type (Form)	Target Ocular Surface Disease/Indication	Clinical Phase/Trial ID	Sponsor/Country
TN-001 Ophthalmic Solution	Transforming Growth Factor Beta 3 (TGF-β3) & Dexamethasone Sodium Phosphate	Viscoelastic biopolymer stabilization vehicle (Gellan/Cellulose-based)	In situ stabilizing topical eye drops	Progressive Keratoconus & Corneal stromal regeneration/stiffening	Phase 1/2/NCT07388069 (Active, Estimated 2026/2027)	TheiaNova Ltd./New Zealand
Lacrimera^®^ Pain & Regeneration Platform	Chitosan-N-acetylcysteine (C-NAC) acting as chemical platform	Thiolated Chitosan (Thiomer)	Electrostatic mucoadhesive hydrogel eye drops	Corneal abrasions, epithelial wounds, and post-surgical surface pain	Clinical Evaluation/NCT05049642 & NCT05064189 (Conducted 2021–2023)	Vienna Institute for Research in Ocular Surgery/Austria
Dexamethasone-Cyclodextrin NP (DECEDE)	Dexamethasone	Cyclodextrin (Starch-derived cyclic oligosaccharide)	Mucopenetrating nanogel/Nanoparticle suspension	Ocular surface-to-posterior segment topical drug delivery platform	Phase 2/NCT01523314 (Evaluated post-2020 for surface kinetics)	King Saud University/Saudi Arabia
SH-XG Dual-Biopolymer Carrier	Glycine & Betaine (Active osmoprotectants for cellular repair)	Hyaluronic Acid (HA) & Xanthan Gum (Interpenetrating Network)	Shear-thinning in situ biopolymer network (Eye drops)	Severe keratitis lesions & Corneal epithelial micro-abrasions	Prospective Multicenter Investigation/NCT05778942 (Published 2025)	Multi-institutional Collaboration/Spain & Italy
OTX-DED Extended Surface Platform	Dexamethasone	Bioresorbable hydrogel matrix (PEG-based)	Intracanalicular hydrogel insert (Continuous tear-film elution)	Ocular surface inflammation & Chronic Dry Eye disease flare-ups	Phase 3/4/NCT05814757	Ocular Therapeutix/USA
KPI-012 (Kala Bio Platform)	Mesenchymal Stem Cell (MSC) Secretome (Growth factors & neurotrophic proteins)	Sodium hyaluronate-integrated biomimetic polymer vehicle	Bioadhesive molecular-stabilizing polymer eye drops	Neurotrophic keratitis & Persistent Corneal Epithelial Defects (PCED)	Phase 2/NCT05727878	Kala Bio/USA
OCS-01 (OPTireach^®^ Technology)	Dexamethasone	Cyclodextrin (Starch-derived cyclic oligosaccharide polymer)	Mucopenetrating polymeric nanoparticle suspension	Postoperative inflammation/pain after cataract surgery & Corneal edema	Phase 3/NCT05147233	Oculis/Switzerland
OTX-TP (Long-acting Insert)	Travoprost	Poly(ethylene glycol) [PEG]-based bioresorbable hydrogel	Intracanalicular hydrogel insert (Slow-release surface elution)	Open-angle glaucoma & Ocular hypertension	Phase 3/NCT04061044	Ocular Therapeutix/USA
EGP-437 (EyeGate System)	Dexamethasone Phosphate	Cross-linked Carboxymethyl Hyaluronate (CMHA) hydrogel	Bioresorbable iontophoretic hydrogel matrix/Topical shield	Non-infectious anterior uveitis & Corneal wound healing acceleration	Phase 3/NCT02517619	EyeGate Pharmaceuticals/USA
APP13007 (APNT Platform)	Clobetasol Propionate (0.05%)	Biocompatible polymeric surfactant matrix (Poloxamer/PVP grid)	Ultra-fine polymer nanoparticle suspension	Ocular surface inflammation and pain post-cataract surgery	Phase 3/NCT04810962	Formosa Pharmaceuticals/Taiwan
TRS01 (Tarsier Micelle)	TRS Immunomodulator compound	Self-assembled amphiphilic block-copolymer (PEG-b-PCL or equivalent)	Polymeric micellar nanogel suspension	Non-infectious anterior uveitis & Ocular surface immune lesions	Phase 3/NCT04222725	Tarsier Pharma/Israel

## Data Availability

The original contributions presented in this study are included in the article. Further inquiries can be directed to the corresponding author.
